# Dietary Niacin and Open-Angle Glaucoma: The Korean National Health and Nutrition Examination Survey

**DOI:** 10.3390/nu10040387

**Published:** 2018-03-22

**Authors:** Kyoung In Jung, Yong Chan Kim, Chan Kee Park

**Affiliations:** Department of Ophthalmology, Seoul St. Mary’s Hospital, College of Medicine, The Catholic University of Korea, 222 Banpo-daero, Seocho-ku, Seoul 137-701, Korea; ezilean@hanmail.net (K.I.J.); mychan2265@gmail.com (Y.C.K.)

**Keywords:** glaucoma, niacin, normal tension glaucoma, nutrients

## Abstract

Glaucoma is a leading cause of loss of sight. High intraocular pressure (IOP) is the most critical risk factor. However, glaucoma develops even within a normal IOP range. Normal tension glaucoma (NTG) is more common in Asia, whereas high tension glaucoma is more common in Western countries. The pathogenesis of glaucoma, especially NTG, is poorly understood. We evaluated the correlation between dietary nutrient intake and glaucoma using data from subjects ≥40 years old from the ongoing, nationwide, population-based study, the Korean National Health and Nutrition Examination Survey V (2008–2012). Dietary intake was determined using the 24 h recall method. Fiber (g/day), ash (g/day), calcium (mg/day), phosphorus (mg/day), iron (mg/day), sodium (mg/day), potassium (mg/day), β-Carotene (μg/day), retinol (μg/day), vitamin A (μg Retinol Equivalents/day), thiamine (mg/day), riboflavin (mg/day), niacin (mg/day), and vitamin C (mg/day) were included in nutrient intake data. All nutrient intake was divided into quartiles. The mean IOP did not differ according to quartiles from any nutrients (all *p* > 0.05). After adjusting for age, gender, income status, education level, smoking, alcohol consumption, physical activity, diabetes, hypertension, IOP, and total energy, the intake of niacin was associated with glaucoma (*p* = 0.013). Among subjects with IOP ≤ 21 mmHg, only niacin was related to glaucoma in a multivariate analysis (*p* = 0.022). Dietary nutrient intake was associated with open-angle glaucoma independent of IOP. Individuals with NTG showed lower intake of niacin among nutrients. This finding suggests the possibility that proper diet counseling may be another modifiable factor, aside from IOP, particularly among patients with NTG.

## 1. Introduction

Glaucoma is a leading cause of loss of sight, affecting more than 70 million people worldwide [1,2]. The pathogenesis of glaucoma is not fully established, although intraocular pressure (IOP) is the most critical and treatable risk factor. Glaucoma develops even within a normal IOP range and can progress, even with a substantial decrease in IOP. Excitotoxicity, unstable blood flow and oxidative stress can also affect the development and progression of glaucoma [3].

A family history of glaucoma is one of the risk factors for primary open-angle glaucoma (POAG) [4]. Family members have a high probability of having common genetic susceptibility or similar lifestyles, such as diet. Given those findings, different genes or environmental factors may be linked to the development of glaucoma. To date, identified gene mutations are responsible for <10% of POAG in the general population [5]. An active interest in environmental or lifestyle factors, such as diet patterns, and their association with glaucoma may be needed to understand the pathogenesis of glaucoma.

The most common type of open-angle glaucoma in Asian countries is normal tension glaucoma (NTG), whereas high tension glaucoma is more common in the United States and Europe [6]. Staple foods and dietary habits are distinctly different between Asian and Caucasian individuals, even though the prevalence of a Westernized diet has increased in Asian countries. The proportion of NTG in a Japanese American population was 80.4% of all POAG cases, which was lower than 92.3% in the Tajimi Study conducted in Japan. One of the reasons for the lower rate of NTG in the Japanese American population may be differences in environmental factors such as nutrition [7,8].

Accumulating clinical evidence suggests a possible association between nutrition or diet composition and POAG [9,10,11,12,13], although a few studies did not find this correlation [14,15]. Lower ingestion of retinol equivalents, vitamins B2 and C, a higher ratio of *n*-3 to *n*-6 polyunsaturated fats, and a lower ingestion of certain fruits or vegetables have been found to be related to the risk for POAG [9,10,11,12,13]. Among nutrients, vitamins with anti-oxidant activity are of interest, as they may relieve oxidative stress in the pathogenesis of glaucoma [16,17].

To date, no study has addressed the relationship between diverse dietary nutrients and glaucoma in Asian countries. In this study, we investigated the potential impact of dietary nutrients on glaucoma using the Korean National Health and Nutrition Examination Survey (KNHANES), a large population-based study. Determination of modifiable risk factors, aside from IOP, can provide other options for treating glaucoma patients showing progression of the disease, despite a relatively low IOP range.

## 2. Methods

The KNHANES is an ongoing nationwide epidemiological study conducted by the Division of Chronic Disease Control and Prevention, Ministry of Health and Welfare, with the approval of its Institutional Review Board. The survey follows the tenets of the Declaration of Helsinki for biomedical research involving humans. Written informed consent was provided by all participants. The KNHANES uses a stratified, multistage, probability cluster survey with a rolling survey model. All participants are randomly chosen from randomly assigned districts of cities and provinces in South Korea.

Among the 37,982 participants in the KNHANES 2008–2012 IV–V, those who were younger than 40 years old or who did not complete an ophthalmic examination were excluded. Other exclusion criteria were as follows: participants with a narrow angle using the Van Herick method (peripheral anterior chamber depth ≤4/1 of peripheral corneal thickness); those whose fundus photograph displayed geographic atrophy or signs of wet age-related macular degeneration, such as retinal pigment epithelial (RPE) detachment, serous detachment of the sensory retina, sub-retinal or sub-RPE hemorrhage, or sub-retinal fibrous scars; those who had not completed the nutritional survey; and those with missing data.

Data on anthropometrics and demographic characteristics, including socioeconomic status and levels of education and physical activity, were collected. Participants were divided into nondrinkers, mild drinkers (≤one time per month), moderate drinkers (>one time and ≤four times per month) and heavy drinkers (>four times per month). Smoking status was classified into nonsmokers, former smokers, and current smokers. Physical activity (PA) scores were categorized based on the International PA Questionnaire guidelines. Moderate physical activity was defined as the performance of moderate-intensity physical activity for ≥20 min 3–4 times/week. The definition of moderate intensity was exercise that induced a mild elevation in breathing or heart rate for at least 10 min. Data on medical comorbidities, such as diabetes mellitus and hypertension, were also collected as potential confounding variables. Diabetes was defined as a fasting blood glucose level higher than 126 mg/dL or the current use of a systemic antidiabetic treatment. Systemic hypertension was defined as a systolic blood pressure (BP) > 160 mmHg, diastolic BP > 90 mmHg, or the current use of a systemic antihypertensive medication.

### 2.1. Ophthalmologic Measurements

Participants underwent ophthalmological examinations, including slit-lamp examinations and fundus photography. Ophthalmologists measured IOP using a Goldmann applanation tonometer (Haag-streit, Inc., Bern, Switzerland).

Participants with elevated IOP (≥22 mmHg) or a glaucomatous optic disc were examined using frequency-doubling technology (Humphrey Matrix; Carl Zeiss Meditec, Inc., Jena, Germany) using the N-30-1 program. A glaucomatous optic disc refers to any of the following: a horizontal or vertical cup-to-disc ratio (vCDR) of ≥0.5, the appearance of optic disc hemorrhage, the presence of a retinal nerve fiber layer defect (RNFL), or a violation of the ISNT rule (neuroretinal rim thickness in the order of inferior > superior > nasal > temporal). Subjects underwent the test again when fixation errors or false-positive errors were >33%. A glaucoma diagnosis was made using modified ISGEO criteria [18,19]: when both fixation errors and false-positive errors were 33% or less, category 1 requires a glaucomatous visual field defect with a CDR of ≥0.7, asymmetry of a vCDR of ≥0.2, or the presence of an RNFL defect [18,19]. When the visual field was not available or was unsatisfactory, subjects were included in category 2 if their vCDR was ≥0.9, the vCDR asymmetry was ≥0.3 or if they had an RNFL loss with a violation of the ISNT rule [19]. If examinations of the optic disc and visual field tests were not possible, category 3 required a visual acuity of <3/60 and IOP exceeding 21 mmHg [18]. Subgroup analysis was done within subjects with NTG; NTG was defined as an IOP was ≤21 mmHg.

### 2.2. Assessment of Nutrient Intake

Dietary intake was determined using the 24 h recall method by trained staff. All subjects were instructed to continue their ordinary diets before the dietary evaluation. The information gained on holidays or weekends was not excluded. Nutrient intake was calculated on the basis of the nutrient concentrations in foods using the Korean Food Composition Table, which was devised by the Korean National Rural Resources Development Institute [20]. Fiber (g/day), ash (g/day), calcium (mg/day), phosphorus (mg/day), iron (mg/day), sodium (mg/day), potassium (mg/day), β-Carotene (μg/day), retinol (μg/day), thiamine (mg/day), riboflavin (mg/day), niacin (mg/day), and Vitamin C (mg/day) were included in nutrient intake data. vitamin A (μg Retinol Equivalents (RE)/day) was calculated by summing retinol (μg/day) and β-Carotene/6 (μg/day). The individuals were divided into quartiles of total intake per day for each nutrient (Q1, Q2, Q3 and Q4). If individuals had taken any kind of supplements consecutively more than 2 weeks over the past year or more than one time per week over the past month, they were regarded as taking supplements.

### 2.3. Statistical Analyses

SAS Software (version 9.2; SAS Institute, Inc., ‎Cary, NC, USA) was used for statistical analyses to reflect sampling weights and to offer nationally representative prevalence estimates. Demographic factors between subjects with and without glaucoma were compared using Student’s *t*-tests for continuous parameters and *χ*^2^ tests for categorical variables. Continuous and categorical parameters are described as the mean ± standard error and percentage, respectively. Multivariate adjusted logistic analysis was performed to investigate factors associated with glaucoma. We analyzed the subset of data after excluding subjects taking supplements. Factors with a difference of *p* < 0.1 between the two groups, IOP, and total energy were entered into a multivariate analysis. Odds ratios (ORs) and 95% confidence intervals were calculated. A *p* value of <0.05 was considered statistically significant.

## 3. Results

**Figure 1:**
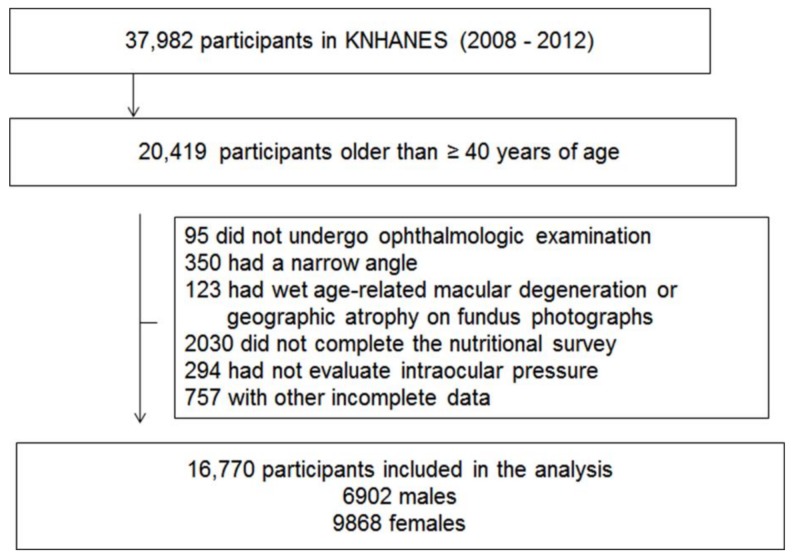
Flow diagram displaying selection of study subjects.

Among 37,982 participants in the KNHANES 2008–2012 IV–V, those younger than 40 years old were excluded (*n* = 17,563). Subjects who did not receive an ophthalmic examination were also excluded (*n* = 95). Participants with a narrow angle using the Van Herick method (peripheral anterior chamber depth ≤4/1 of peripheral corneal thickness; *n* = 350), those whose fundus photograph displayed geographic atrophy or signs of wet age-related macular degeneration, such as retinal pigment epithelial (RPE) detachment, serous detachment of the sensory retina, sub-retinal or sub-RPE hemorrhage, or sub-retinal fibrous scars (*n* = 123), those who had not completed the nutritional survey (*n* = 2030), and those with missing data (*n* = 757) were excluded. A total 16,770 participants (6902 men, 9868 women) were included in this study (Figure 1).

The demographics are shown in Table 1. Seven hundred and seventy-five participants were diagnosed with glaucoma (overall prevalence, 4.11%). The proportion of individuals with glaucoma diagnosis made in the absence of visual field defects and/or abnormal optic disc parameters was 47.4%. The average age of participants with glaucoma was 60.7 ± 0.6 years old. Subjects with glaucoma were older and less educated, exercised less regularly, were more likely to smoke, have no occupation, and have diabetes or hypertension than those without glaucoma (all *p* < 0.05). IOP was higher in subjects with glaucoma (14.7 ± 0.2 mmHg) than in those without glaucoma (14.0 ± 0.1 mmHg, *p* < 0.001).

Quartile categories of each nutrient intake are displayed in Table 2.

With regard to nutrients, intake of crude fiber, ash, calcium, phosphorus, iron, sodium, potassium, vitamin A, β-Carotene, retinol, thiamin, riboflavin, niacin, and vitamin C was associated with glaucoma (all *p* < 0.05; Table 3).

Multivariate analyses (model 1) adjusted for age, gender, income status, education level, smoking, alcohol consumption, physical activity, diabetes, hypertension, IOP, and total energy, showed intake of niacin was correlated with glaucoma (*p* = 0.013; Table 4). In a partially adjusted model (all covariates in model 1 but IOP), overall results were similar to those in model 1 multivariate analyses.

IOP distributions by nutrient quartiles are displayed in Table 5. The mean IOP did not differ according to nutrient quartiles.

Subgroup analyses showed that for subjects with IOP ≤ 21 mmHg (*n* = 767, 98.9% of total glaucoma patients), only lower niacin intake was associated with a higher odds ratio for glaucoma (*p* = 0.022; Table 6).

After the exclusion of individuals taking supplements, higher intake of riboflavin (*p* = 0.009) and niacin (*p* = 0.035) were related to a lower risk of glaucoma (Table 7). Among subjects without glaucoma, 4100 subjects (25.6%) were taking supplements. Among individuals with glaucoma, 193 individuals (24.9%) were taking supplements.

## 4. Discussion

We demonstrated that dietary intake of niacin was associated with glaucoma, independent of IOP. Mean IOP was similar by quartiles of all examined nutrients. Individuals with NTG showed lower intake of niacin among nutrients. After the exclusion of individuals taking supplements, higher intake of riboflavin and niacin were related to a lower risk of glaucoma. Overall, lower intake of niacin remained significantly associated with glaucoma also in the subgroup analysis.

Previously, some reports found that low intake of vitamins was associated with augmented risk for glaucoma [11,13]. The Rotterdam study, a prospective population-based study, reported that a low intake of retinol equivalents and vitamin B1 appeared to increase the risk for POAG [11]. Wang et al. suggested that supplementary ingestion of vitamin C was associated with reduced glaucoma risk [13]. The Nurses’ Health Study and Health Professionals Follow-up Study did not detect a correlation between nutrients with anti-oxidant properties and open-angle glaucoma [14]. However, the diagnosis of glaucoma was based on self-report, even though a strength of that study was that it was a large prospective study [14]. This could result in a selection bias because over half of individuals with glaucoma are not aware of their condition [21,22].

IOP is the most critical risk factor for glaucoma, even though glaucoma can develop in cases with a normal range of IOP. We analyzed IOP distribution by quartiles of dietary nutrients and found no significant differences in mean IOP according to dietary nutrients. The relationship between nutrients and glaucoma was analyzed adjusting for IOP to remove the influence of dietary nutrient intake on IOP. There was an association between dietary intake of niacin with glaucoma, independent of IOP.

With regard to NTG, only one Japanese study observed that lower *serum* vitamin C levels were correlated with increased risk of NTG [23]. The authors speculated that glutamate-stimulated release of vitamin C might decrease oxidative stress induced by glutamate excitotoxicity and reduce the degeneration of retinal ganglion cells [23]. The difference between that study and this study is that only vitamin A, B9, C, E and uric acid were investigated using *serum* samples, and niacin was not included in that study [23].

In this study, only dietary niacin intake was lower in subjects with NTG. In addition, niacin remained as the factor associated with glaucoma after the exclusion of subjects taking supplements. Recently, Williams et al. found that oral administration of vitamin B3 protected retinal ganglion cells in aged mouse with chronic ocular hypertension, modulating mitochondrial vulnerability [24]. In a stroke animal model, niacin (nicotinic acid) treatment promoted synaptic plasticity and axon growth [25]. That study suggested that brain-derived neurotrophic factor (BDNF)/tropomyosin receptor kinase B (TrKB) pathways seemed to be involved in niacin-induced neuroprotective effects after a stroke [25]. Disrupted axonal transport of neurotrophic factors is one of main mechanisms of glaucoma [3]. Therefore, upregulated BDNF by niacin treatment might decrease the risk of glaucoma. Kaplon et al. reported that niacin intake was positively correlated with better vascular endothelial function associated with decreased vascular oxidative stress [26]. The vascular hypothesis for glaucoma pathogenesis states insufficient or unstable blood supply is a key contributing factor for glaucoma development or progression [27]. A collaborative normal tension glaucoma study showed vascular factors, such as disc hemorrhage and migraine, were risk factors for the progression of glaucoma [28]. Compromised vascular endothelial cell function has been reported in subjects with NTG [29,30]. Therefore, it appears that improved vascular endothelial cell function induced by niacin could lower the risk for open-angle glaucoma. To the best of our knowledge, this is the first study to reveal the relationship between niacin and glaucoma in human. However, the study was simply descriptive and inconclusive on disease mechanisms. Measurement of serum or intravitreal level of niacin in glaucoma patients would be helpful to understand the mechanism of how low niacin influences glaucoma.

The strength of this population-based study is that study participants were representative of subjects with glaucoma in the general population. We present the first study analyzing the relationship between dietary nutrients and glaucoma (especially NTG) in an Asian country. One limitation of this study is that we could not determine cause and effect for the relationship between dietary nutrients and glaucoma. Prospective randomized controlled trial or epidemiological cohort studies are needed to ensure the usefulness of niacin in glaucoma. Another limitation is that the nutritional survey was conducted just once, although all subjects were educated to continue their ordinary diets before the dietary evaluation. There might be a confounding effect of seasonal foods because all participants did not take part in the nutritional survey at the same time of year. The proportion of participants taking dietary reference intakes for Koreans (KDRIs) was relatively lower for calcium (67.0 ± 0.8%) and potassium (61.9 ± 0.6%), according to data from the Korea National Health and Nutrition Examination Survey (2012). Therefore, the association of glaucoma with the intake of these nutrients might be underestimated. Nutrient intake was calculated on the basis of the nutrient concentrations in foods using the Korean Food Composition Table. Potential errors can exist in the tables that describe foods. The errors may result in inaccurate measurement of nutrient intake at the individual level, though probably less so at the group level. The Korean Food Composition Tables cover nearly 3000 food items in 19 groups, being almost complete for nutritional evaluation of daily foods. Kim et al. reported the accuracy of conventional food composition table-based estimation of intakes of protein, lipid and carbohydrate, in comparison with chemical analysis [31]. Their result supports that the Korean Food Composition Tables are sufficiently accurate, even though that study did not evaluate the accuracy of niacin. Analysis from dietary nutrients has limitations because the bioavailability of nutrients may vary in each individual. Further, serum analysis of nutrients may be needed to investigate the direct association of glaucoma and nutrients.

## 5. Conclusions

In conclusion, this population-based study found that lower nutrient intake of niacin was associated with glaucoma. Lower niacin intake was associated with NTG. In addition, niacin remained as the factor related to glaucoma after the exclusion of subjects taking supplements. Glaucoma is a progressive neurodegenerative disease and can lead to vision loss, despite a substantial decrease in IOP. The prevention of glaucoma is important, because neurodegeneration of retinal ganglion cells is irreversible. Dietary nutrition is a modifiable factor; the discovery that nutrition can help decrease glaucoma development or progression may help individuals with glaucoma even though this needs confirmation from longitudinal, prospective studies.

## Figures and Tables

**Table 1:** Baseline characteristics.

Characteristics	Glaucoma		*p* Value
	No	Yes	
	*n* = 15995	*n* = 775	
Age (years)	55.2 ± 0.2	60.7 ± 0.6	<0.001
Gender			
Male	6552 (47.83)	350 (54.36)	0.006
Female	9443 (52.17)	425 (45.64)	
BMI (kg/m^2^)			
Underweight (<18.5)	492 (2.78)	35 (4.18)	0.108
Normal weight (≥18.5, <23)	5825 (35.69)	296 (37.78)	
Overweight (≥23, <25)	4137 (25.86)	198 (26.64)	
Obese (≥25)	5541 (35.67)	246 (31.40)	
Income status			
Lowest	4155 (20.39)	296 (29.25)	<0.001
Lower middle	3985 (25.60)	183 (24.31)	
Higher middle	3794 (26.20)	136 (21.69)	
Highest	4061 (27.80)	160 (24.75)	
Education			
Elementary school or less	6199 (30.74)	409 (44.64)	<0.001
Middle school graduate	2466 (15.65)	98 (12.31)	
High school graduate	4503 (32.79)	161 (24.42)	
University graduate or higher	2827 (20.81)	107 (18.63)	
Occupation			
White collar	2047 (16.55)	53 (9.71)	<0.001
Blue collar	7142 (47.74)	323 (44.42)	
Inoccupation	6806 (35.71)	399 (45.87)	
Smoking			
Never	9760 (54.90)	437 (48.41)	0.015
Former	3961 (29.25)	192 (31.19)	
Current	2274 (15.85)	146 (20.41)	
Alcohol intake (n)			
Non-drink	5705 (30.22)	327 (35.28)	0.064
≤1 in month	4322 (26.72)	194 (25.44)	
≤4 in month	2697 (18.75)	102 (14.85)	
≥2 in week	3271 (24.32)	152 (24.44)	
Region of residence			
Rural	6134 (33.24)	329 (35.37)	0.356
Urban	9861 (66.76)	446 (64.63)	
Physical activity (≥moderate intensity)			
No	12,473 (77.72)	632 (81.48)	0.047
Yes	3522 (22.28)	143 (18.52)	
Diabetes			
No	13,798 (87.43)	612 (79.88)	<0.001
Yes	2197 (12.57)	163 (20.12)	
Hypertension			
No	9638 (64.21)	370 (49.72)	<0.001
Yes	6357 (35.79)	405 (50.28)	
Intraocular pressure (mmHg)	14.0 ± 0.1	14.7 ± 0.2	<0.001

Data are presented as mean ± SE, *n* (weighted %). Statistics were carried out using *t*-test, Rao-Scott Chi-square.

**Table 2:** Quartile categories of nutrient intake.

Nutrient	Q1	Q2	Q3	Q4
Total energy intake (kcal)	<1387.6	≥1387.6	≥1814.5	≥2352.1
Crude fiber (g/day)	<4.54	≥4.54	≥6.77	≥9.72
Ash (g/day)	<12.53	≥12.53	≥18.13	≥25.30
Calcium (mg/day)	<283.08	≥283.08	≥436.92	≥645.29
Phosphorus (mg/day)	<807.97	≥807.97	≥1083.58	≥1444.31
Iron (mg/day)	<8.63	≥8.63	≥12.90	≥19.00
Sodium(mg/day)	<2731.53	≥2731.53	≥4239.08	≥6368.29
Potassium (mg/day)	<2005.32	≥2005.32	≥2824.62	≥3884.43
Vitamin A (μg Retinol Equivalents/day)	<316.68	≥316.68	≥602.05	≥1050.90
β-Carotene (μg/day)	<1556.89	≥1556.89	≥3103.72	≥5586.47
Retinol (μg/day)	<8.45	≥8.45	≥41.02	≥105.74
Thiamin (mg/day)	<0.78	≥0.78	≥1.11	≥1.57
Riboflavin (mg/day)	<0.67	≥0.67	≥1.03	≥1.49
Niacin (mg/day)	<10.15	≥10.15	≥14.42	≥20.49
Vitamin C (mg/day)	<50.48	≥50.48	≥85.71	≥141.21

**Table 3:** The association between dietary nutrient intakes and glaucoma.

Nutrient	Gender = Male		*p* Value	Nutrient	Gender = Female		*p* Value
	Glaucoma				Glaucoma		
	No	Yes			No	Yes	
	*n* = 6552	*n* = 350			*n* = 9443	*n* = 425	
Energy intake				Energy intake			
Q1	815 (10.54)	70 (16.87)	0.002	Q1	3600 (38.05)	178 (40.00)	0.130
Q2	1469 (19.86)	89 (25.66)		Q2	2814 (29.36)	151 (33.68)	
Q3	1907 (28.95)	92 (23.43)		Q3	2033 (21.69)	69 (18.65)	
Q4	2361 (40.65)	99 (34.04)		Q4	996 (10.90)	27 (7.66)	
Crude fiber				Crude fiber			
Q1	1208 (17.25)	70 (21.55)	0.368	Q1	3014 (31.70)	165 (39.68)	0.009
Q2	1597 (23.80)	83 (24.28)		Q2	2420 (26.06)	113 (27.20)	
Q3	1807 (28.43)	90 (26.67)		Q3	2070 (22.02)	81 (18.74)	
Q4	1940 (30.53)	107 (27.50)		Q4	1939 (20.22)	66 (14.38)	
Ash				Ash			
Q1	1107 (14.07)	79 (22.45)	0.003	Q1	3362 (34.44)	190 (43.36)	0.011
Q2	1512 (21.62)	77 (19.40)		Q2	2641 (28.29)	114 (26.32)	
Q3	1795 (28.28)	97 (29.71)		Q3	2047 (21.96)	74 (20.27)	
Q4	2138 (36.03)	97 (28.44)		Q4	1393 (15.30)	47 (10.05)	
Calcium				Calcium			
Q1	1258 (15.97)	82 (25.02)	0.004	Q1	3231 (32.70)	175 (40.03)	0.022
Q2	1586 (24.11)	88 (20.81)		Q2	2434 (25.90)	106 (27.90)	
Q3	1774 (28.41)	77 (21.55)		Q3	2047 (22.32)	76 (17.09)	
Q4	1934 (31.51)	103 (32.62)		Q4	1731 (19.07)	68 (14.98)	
Phosphorus				Phosphorus			
Q1	985 (12.69)	70 (19.80)	0.019	Q1	3480 (35.78)	194 (44.55)	0.006
Q2	1467 (20.87)	89 (22.29)		Q2	2708 (28.80)	118 (27.51)	
Q3	1865 (28.56)	91 (25.07)		Q3	2009 (21.93)	78 (19.82)	
Q4	2235 (37.88)	100 (32.83)		Q4	1246 (13.48)	35 (8.12)	
Iron				Iron			
Q1	1235 (16.00)	84 (25.42)	0.004	Q1	3216 (32.57)	179 (42.70)	0.004
Q2	1555 (23.38)	80 (19.26)		Q2	2456 (26.67)	108 (26.11)	
Q3	1783 (28.56)	90 (27.55)		Q3	1997 (21.91)	75 (17.85)	
Q4	1979 (32.06)	96 (27.76)		Q4	1774 (18.85)	63 (13.33)	
Sodium				Sodium			
Q1	1085 (13.85)	73 (18.58)	0.019	Q1	3418 (35.00)	171 (38.55)	0.584
Q2	1499 (21.47)	69 (19.72)		Q2	2669 (28.31)	120 (28.51)	
Q3	1833 (28.65)	112 (33.91)		Q3	1974 (21.51)	78 (18.91)	
Q4	2135 (36.03)	96 (27.78)		Q4	1382 (15.18)	56 (14.03)	
Potassium				Potassium			
Q1	1154 (15.07)	76 (22.76)	0.014	Q1	3266 (33.48)	188 (44.12)	<0.001
Q2	1564 (22.70)	83 (22.04)		Q2	2487 (27.10)	123 (28.56)	
Q3	1827 (28.79)	99 (29.10)		Q3	2027 (21.66)	65 (15.86)	
Q4	2007 (33.43)	92 (26.10)		Q4	1663 (17.76)	49 (11.46)	
Vitamin A				Vitamin A			
Q1	1466 (19.27)	94 (25.08)	0.150	Q1	2922 (29.68)	171 (40.06)	<0.001
Q2	1570 (24.15)	82 (25.32)		Q2	2439 (25.72)	108 (25.86)	
Q3	1745 (27.53)	84 (24.42)		Q3	2094 (22.85)	92 (21.16)	
Q4	1771 (29.05)	90 (25.18)		Q4	1988 (21.74)	54 (12.92)	
β-Carotene				β-Carotene			
Q1	1472 (19.69)	91 (23.99)	0.247	Q1	2876 (29.32)	173 (40.70)	<0.001
Q2	1577 (24.29)	83 (26.92)		Q2	2387 (25.61)	101 (23.44)	
Q3	1724 (27.12)	85 (23.53)		Q3	2176 (23.29)	89 (20.65)	
Q4	1779 (28.89)	91 (25.56)		Q4	2004 (21.78)	62 (15.22)	
Retinol				Retinol			
Q1	1551 (19.54)	104 (24.27)	0.035	Q1	3024 (29.58)	173 (37.05)	0.004
Q2	1728 (26.03)	101 (30.78)		Q2	2266 (23.68)	108 (27.10)	
Q3	1564 (24.81)	85 (22.90)		Q3	2292 (25.37)	87 (22.49)	
Q4	1709 (29.62)	60 (22.05)		Q4	1861 (21.36)	57 (13.36)	
Thiamin				Thiamin			
Q1	1117 (14.11)	77 (19.68)	0.074	Q1	3368 (34.49)	190 (44.33)	0.002
Q2	1535 (22.04)	95 (25.03)		Q2	2569 (27.60)	125 (28.17)	
Q3	1779 (27.85)	88 (24.40)		Q3	2109 (22.69)	67 (17.41)	
Q4	2121 (36.00)	90 (30.89)		Q4	1397 (15.22)	43 (10.09)	
Riboflavin				Riboflavin			
Q1	1275 (15.92)	89 (24.31)	0.005	Q1	3260 (32.59)	200 (45.37)	<0.001
Q2	1545 (21.93)	93 (24.04)		Q2	2646 (27.78)	117 (27.17)	
Q3	1709 (27.34)	86 (24.57)		Q3	2056 (23.26)	60 (14.76)	
Q4	2023 (34.81)	82 (27.08)		Q4	1481 (16.38)	48 (12.70)	
Niacin				Niacin			
Q1	1037 (13.17)	75 (20.94)	0.007	Q1	3471 (35.13)	217 (48.88)	<0.001
Q2	1518 (21.49)	89 (23.47)		Q2	2657 (28.18)	115 (27.81)	
Q3	1798 (27.42)	101 (25.91)		Q3	2083 (23.08)	61 (15.88)	
Q4	2199 (37.92)	85 (29.69)		Q4	1232 (13.61)	32 (7.44)	
Vitamin C				Vitamin C			
Q1	1445 (20.03)	83 (22.78)	0.360	Q1	2812 (29.17)	159 (37.95)	0.008
Q2	1662 (24.72)	90 (27.81)		Q2	2352 (25.10)	114 (25.58)	
Q3	1748 (27.91)	94 (26.41)		Q3	2100 (22.50)	84 (19.02)	
Q4	1697 (27.35)	83 (23.00)		Q4	2179 (23.23)	68 (17.46)	

Data are presented as *n* (weighted %). Statistics were carried out using *t*-test, Rao-Scott Chi-square test.

**Table 4:** Odds ratios of nutritional factors for glaucoma.

Nutrient	Unadjusted		Model 1		Nutrient	Unadjusted		Model 1	
	OR (95% CI)	*p* for Trend	OR (95% CI)	*p* for Trend		OR (95% CI)	*p* for Trend	OR (95% CI)	*p* for Trend
Crude fiber					Vitamin A				
Q1	Reference		Reference		Q1	Reference		Reference	
Q2	0.85 (0.67–1.08)	0.005	0.96 (0.74–1.23)	0.221	Q2	0.79 (0.63–1.01)	<0.001	0.99 (0.78–1.26)	0.130
Q3	0.76 (0.59–0.98)		0.90 (0.69–1.17)		Q3	0.71 (0.55–0.91)		0.93 (0.70–1.22)	
Q4	0.71 (0.55–0.91)		0.85 (0.63–1.13)		Q4	0.60 (0.46–0.78)		0.81 (0.61–1.07)	
Ash					β-Carotene				
Q1	Reference		Reference		Q1	Reference		Reference	
Q2	0.69 (0.54–0.89)	0.001	0.82 (0.62–1.07)	0.369	Q2	0.79 (0.62–1.02)	<0.001	0.97 (0.76–1.24)	0.220
Q3	0.79 (0.61–1.01)		0.95 (0.72–1.27)		Q3	0.69 (0.53–0.90)		0.89 (0.68–1.17)	
Q4	0.61 (0.47–0.80)		0.79 (0.55–1.14)		Q4	0.65 (0.50–0.83)		0.85 (0.65–1.12)	
Calcium					Retinol				
Q1	Reference		Reference		Q1			Reference	
Q2	0.74 (0.58–0.95)	0.020	0.89 (0.68–1.16)	0.615	Q2	0.97 (0.76–1.22)	<0.001	1.14 (0.90–1.44)	0.192
Q3	0.60 (0.46–0.78)		0.75 (0.56–0.99)		Q3	0.75 (0.59–0.95)		0.99 (0.76–1.27)	
Q4	0.76 (0.58–1.00)		0.98 (0.72–1.33)		Q4	0.59 (0.45–0.78)		0.84 (0.63–1.13)	
Phosphorus					Thiamin				
Q1	Reference		Reference		Q1	Reference		Reference	
Q2	0.79 (0.62–1.00)	0.006	0.87 (0.66–1.14)	0.439	Q2	0.85 (0.66–1.09)	0.002	0.97 (0.74–1.28)	0.366
Q3	0.72 (0.56–0.93)		0.85 (0.62–1.17)		Q3	0.67 (0.52–0.88)		0.84 (0.61–1.15)	
Q4	0.68 (0.51–0.91)		0.86 (0.55–1.33)		Q4	0.68 (0.51–0.90)		0.88 (0.60–1.30)	
Iron					Riboflavin				
Q1	Reference		Reference		Q1	Reference		Reference	
Q2	0.66 (0.52–0.84)	0.001	0.77 (0.59–0.99)	0.075	Q2	0.74 (0.58–0.94)	<0.001	0.88 (0.68–1.14)	0.104
Q3	0.68 (0.52–0.90)		0.81 (0.60–1.10)		Q3	0.58 (0.45–0.75)		0.74 (0.55–1.00)	
Q4	0.62 (0.48–0.82)		0.72 (0.52–1.00)		Q4	0.59 (0.45–0.78)		0.79 (0.54–1.15)	
Sodium					Niacin				
Q1	Reference		Reference		Q1	Reference		Reference	
Q2	0.85 (0.67–1.09)	0.110	1.00 (0.77–1.30)	0.452	Q2	0.75 (0.58–0.95)	<0.001	0.80 (0.61–1.06)	0.013
Q3	0.98 (0.77–1.24)		1.22 (0.94–1.58)		Q3	0.62 (0.48–0.80)		0.69 (0.50–0.95)	
Q4	0.77 (0.59–0.99)		1.04 (0.76–1.43)		Q4	0.57 (0.42–0.76)		0.60 (0.40–0.92)	
Potassium					Vitamin C				
Q1	Reference		Reference		Q1	Reference		Reference	
Q2	0.76 (0.59–0.97)	<0.001	0.88 (0.67–1.16)	0.090	Q2	0.90 (0.70–1.15)	0.004	1.08 (0.84–1.39)	0.422
Q3	0.70 (0.53–0.92)		0.83 (0.61–1.14)		Q3	0.77 (0.59–1.00)		0.98 (0.74–1.28)	
Q4	0.58 (0.44–0.77)		0.72 (0.49–1.05)		Q4	0.68 (0.52–0.89)		0.90 (0.67–1.22)	

Data are presented OR (95% CI). Statistics were carried out using Logistic regression. Model 1 was adjusted for age, gender, income, education, occupation, smoking, drink, physical activity, diabetes, hypertension, intraocular pressure, total energy.CI, confidence interval; OR, odds ratio.

**Table 5:** Intraocular pressure according to quartiles of nutrient intake.

Nutrient	Intraocular Pressure (mmHg)	Nutrient	Intraocular Pressure (mmHg)
Crude fiber		Vitamin A	
Q1	13.95 ± 0.07	Q1	14.05 ± 0.07
Q2	14.00 ± 0.06	Q2	13.94 ± 0.07
Q3	14.08 ± 0.07	Q3	14.01 ± 0.07
Q4	13.97 ± 0.07	Q4	14.00 ± 0.07
*p* for trend	0.532	*p* for trend	0.701
Ash		β-Carotene	
Q1	13.95 ± 0.06	Q1	14.05 ± 0.07
Q2	13.97 ± 0.07	Q2	13.99 ± 0.07
Q3	14.10 ± 0.07	Q3	14.00 ± 0.06
Q4	13.98 ± 0.06	Q4	13.97 ± 0.07
*p* for trend	0.339	*p* for trend	0.400
Ca		Retinol	
Q1	13.98 ± 0.07	Q1	13.90 ± 0.06
Q2	13.99 ± 0.06	Q2	14.03 ± 0.07
Q3	14.07 ± 0.07	Q3	14.07 ± 0.06
Q4	13.96 ± 0.07	Q4	14.01 ± 0.07
*p* for trend	0.914	*p* for trend	0.135
Phosphorus		Thiamin	
Q1	13.95 ± 0.07	Q1	13.92 ± 0.07
Q2	13.97 ± 0.06	Q2	14.01 ± 0.07
Q3	14.06 ± 0.06	Q3	13.99 ± 0.07
Q4	14.03 ± 0.07	Q4	14.09 ± 0.07
*p* for trend	0.186	*p* for trend	0.054
Iron		Riboflavin	
Q1	13.96 ± 0.07	Q1	13.97 ± 0.06
Q2	14.02 ± 0.07	Q2	13.93 ± 0.07
Q3	14.02 ± 0.06	Q3	14.04 ± 0.07
Q4	14.01 ± 0.07	Q4	14.07 ± 0.07
*p* for trend	0.551	*p* for trend	0.089
Sodium		Niacin	
Q1	13.97 ± 0.06	Q1	13.98 ± 0.07
Q2	13.95 ± 0.07	Q2	13.95 ± 0.06
Q3	14.09 ± 0.07	Q3	14.00 ± 0.06
Q4	14.00 ± 0.07	Q4	14.08 ± 0.07
*p* for trend	0.331	*p* for trend	0.195
Potassium		Vitamin C	
Q1	13.96 ± 0.07	Q1	14.01 ± 0.07
Q2	13.99 ± 0.06	Q2	13.95 ± 0.06
Q3	14.07 ± 0.06	Q3	14.01 ± 0.07
Q4	13.98 ± 0.07	Q4	14.03 ± 0.07
*p* for trend	0.621	*p* for trend	0.646

Data are presented as mean ± SE.

**Table 6:** Odds ratios of nutritional factors for glaucoma in subjects with intraocular pressure ≤21 mmHg.

Nutrient	OR (95% CI)	*p* for Trend	Nutrient	OR (95% CI)	*p* for Trend
Crude fiber			Vitamin A		
Q1	Reference		Q1	Reference	
Q2	0.99 (0.77–1.21)	0.311	Q2	1.01 (0.80–1.28)	0.161
Q3	0.92 (0.71–1.21)		Q3	0.94 (0.72–1.24)	
Q4	0.87 (0.66–1.17)		Q4	0.82 (0.62–1.09)	
Ash			β-Carotene		
Q1	Reference		Q1	Reference	
Q2	0.82 (0.62–1.08)	0.438	Q2	0.98 (0.77–1.24)	0.251
Q3	0.98 (0.73–1.30)		Q3	0.89 (0.68–1.18)	
Q4	0.80 (0.56–1.16)		Q4	0.86 (0.66–1.13)	
Calcium			Retinol		
Q1	Reference		Q1	Reference	
Q2	0.91 (0.69–1.19)	0.635	Q2	1.17 (0.92–1.48)	0.210
Q3	0.74 (0.56–0.99)		Q3	0.99 (0.76–1.28)	
Q4	0.99 (0.73–1.35)		Q4	0.86 (0.64–1.15)	
Phosphorus			Thiamin		
Q1	Reference		Q1	Reference	
Q2	0.88 (0.67–1.15)	0.440	Q2	0.98 (0.75–1.30)	0.428
Q3	0.85 (0.62–1.17)		Q3	0.85 (0.62–1.17)	
Q4	0.86 (0.55–1.34)		Q4	0.90 (0.61–1.33)	
Iron			Riboflavin		
Q1	Reference		Q1	Reference	
Q2	0.77 (0.59–0.99)	0.091	Q2	0.91 (0.70–1.18)	0.154
Q3	0.82 (0.61–1.12)		Q3	0.77 (0.57–1.04)	
Q4	0.73 (0.53–1.01)		Q4	0.82 (0.56–1.18)	
Sodium			Niacin		
Q1	Reference		Q1	Reference	
Q2	1.01 (0.77–1.31)	0.364	Q2	0.82 (0.63–1.09)	0.022
Q3	1.25 (0.96–1.62)		Q3	0.71 (0.51–0.99)	
Q4	1.06 (0.78–1.46)		Q4	0.63 (0.41–0.96)	
Potassium			Vitamin C		
Q1	Reference		Q1	Reference	
Q2	0.90 (0.69–1.19)	0.128	Q2	1.10 (0.85–1.41)	0.455
Q3	0.86 (0.62–1.18)		Q3	0.98 (0.74–1.29)	
Q4	0.74 (0.50–1.08)		Q4	0.91 (0.68–1.23)	

Data are presented OR (95% CI). Statistics were carried out using Logistic regression. Odds ratios are adjusted for age, gender, income, education, occupation, smoking, drink, physical activity, diabetes, hypertension, intraocular pressure, total energy.

**Table 7:** Odds ratios of nutritional factors for glaucoma after exclusion of participants taking supplements.

Nutrient	OR (95% CI)	*p* for Trend	Nutrient	OR (95% CI)	*p* for Trend
Crude fiber			Vitamin A		
Q1	Reference		Q1	Reference	
Q2	0.96 (0.73–1.28)	0.410	Q2	0.92 (0.70–1.21)	0.481
Q3	0.88 (0.64–1.20)		Q3	0.95 (0.69–1.30)	
Q4	0.90 (0.65–1.24)		Q4	0.87 (0.63–1.21)	
Ash			β-Carotene		
Q1	Reference		Q1	Reference	
Q2	0.84 (0.61–1.16)	0.377	Q2	0.94 (0.71–1.25)	0.505
Q3	0.91 (0.65–1.27)		Q3	0.89 (0.65–1.22)	
Q4	0.80 (0.53–1.21)		Q4	0.91 (0.67–1.24)	
Calcium			Retinol		
Q1	Reference		Q1	Reference	
Q2	0.91 (0.65–1.26)	0.618	Q2	1.12 (0.85–1.48)	0.125
Q3	0.78 (0.56–1.08)		Q3	0.91 (0.68–1.21)	
Q4	0.96 (0.67–1.37)		Q4	0.82 (0.60–1.13)	
Phosphorus			Thiamin		
Q1	Reference		Q1	Reference	
Q2	0.83 (0.60–1.15)	0.419	Q2	0.92 (0.67–1.25)	0.267
Q3	0.82 (0.56–1.20)		Q3	0.89 (0.62–1.29)	
Q4	0.82 (0.47–1.40)		Q4	0.76 (0.48–1.20)	
Iron			Riboflavin		
Q1	Reference		Q1	Reference	
Q2	0.70 (0.52–0.94)	0.0502	Q2	0.81 (0.60–1.08)	0.009
Q3	0.88 (0.63–1.23)		Q3	0.63 (0.44–0.89)	
Q4	0.64 (0.44–0.91)		Q4	0.60 (0.39–0.93)	
Sodium			Niacin		
Q1	Reference		Q1	Reference	
Q2	1.00 (0.74–1.35)	0.424	Q2	0.73 (0.52–1.02)	0.035
Q3	1.26 (0.94–1.69)		Q3	0.75 (0.51–1.12)	
Q4	1.05 (0.73–1.50)		Q4	0.54 (0.33–0.90)	
Potassium			Vitamin C		
Q1	Reference		Q1	Reference	
Q2	0.70 (0.51–0.96)	0.100	Q2	1.14 (0.86–1.53)	0.726
Q3	0.75 (0.53–1.07)		Q3	1.01 (0.75–1.36)	
Q4	0.68 (0.44–1.04)		Q4	1.11 (0.79–1.55)	

Data are presented OR (95% CI). Statistics were carried out using Logistic regression, adjusted for age, gender, income, education, occupation, smoking, drink, Physical activity, diabetes, hypertension, Intraocular pressure, total energy.

## References

[1] Quigley H.A., Broman A.T. (2006). The number of people with glaucoma worldwide in 2010 and 2020. Br. J. Ophthalmol..

[2] Resnikoff S., Pascolini D., Etya’Ale D., Kocur I., Pararajasegaram R., Pokharel G.P., Mariotti S.P. (2004). Global data on visual impairment in the year 2002. Bull. World Health Org..

[3] Weinreb R.N., Aung T., Medeiros F.A. (2014). The pathophysiology and treatment of glaucoma: A review. JAMA.

[4] Tielsch J.M., Katz J., Sommer A., Quigley H.A., Javitt J.C. (1994). Family history and risk of primary open angle glaucoma: The Baltimore eye survey. Arch. Ophthalmol..

[5] Abu-Amero K., Kondkar A.A., Chalam K.V. (2015). An updated review on the genetics of primary open angle glaucoma. Int. J. Mol. Sci..

[6] Mudumbai R.C. (2013). Clinical update on normal tension glaucoma. Semin. Ophthalmol..

[7] Iwase A., Suzuki Y., Araie M., Yamamoto T., Abe H., Shirato S., Kuwayama Y., Mishima H.K., Shimizu H., Tomita G. (2004). The prevalence of primary open-angle glaucoma in Japanese: The Tajimi Study. Ophthalmology.

[8] Pekmezci M., Vo B., Lim A.K., Hirabayashi D.R., Tanaka G.H., Weinreb R.N., Lin S.C. (2009). The characteristics of glaucoma in Japanese Americans. Arch. Ophthalmol..

[9] Coleman A.L., Stone K.L., Kodjebacheva G., Yu F., Pedula K.L., Ensrud K.E., Cauley J.A., Hochberg M.C., Topouzis F., Badala F. (2008). Glaucoma risk and the consumption of fruits and vegetables among older women in the study of osteoporotic fractures. Am. J. Ophthalmol..

[10] Kang J.H., Pasquale L.R., Willett W.C., Rosner B.A., Egan K.M., Faberowski N., Hankinson S.E. (2004). Dietary fat consumption and primary open-angle glaucoma. Am. J. Clin. Nutr..

[11] Ramdas W.D., Wolfs R.C., Kiefte-de Jong J.C., Hofman A., de Jong P.T., Vingerling J.R., Jansonius N.M. (2012). Nutrient intake and risk of open-angle glaucoma: The Rotterdam Study. Eur. J. Epidemiol..

[12] Renard J.P., Rouland J.F., Bron A., Sellem E., Nordmann J.P., Baudouin C., Denis P., Villain M., Chaine G., Colin J. (2013). Nutritional, lifestyle and environmental factors in ocular hypertension and primary open-angle glaucoma: An exploratory case-control study. Acta Ophthalmol..

[13] Wang S.Y., Singh K., Lin S.C. (2013). Glaucoma and vitamins A, C, and E supplement intake and serum levels in a population-based sample of the United States. Eye. Lond..

[14] Kang J.H. (2003). Antioxidant intake and primary open-angle glaucoma: A prospective study. Am. J. Epidemiol..

[15] Li S., Li D., Shao M., Cao W., Sun X. (2017). Lack of association between serum vitamin B6, vitamin B12, and vitamin D levels with different types of glaucoma: A systematic review and meta-analysis. Nutrients.

[16] Engin K.N. (2009). Alpha-tocopherol: Looking beyond an antioxidant. Mol. Vis..

[17] Ritch R. (2000). Neuroprotection: Is it already applicable to glaucoma therapy?. Curr. Opin. Ophthalmol..

[18] Foster P.J., Buhrmann R., Quigley H.A., Johnson G.J. (2002). The definition and classification of glaucoma in prevalence surveys. Br. J. Ophthalmol..

[19] Kim D.W., Kim Y.K., Jeoung J.W., Kim D.M., Park K.H. (2015). Prevalence of optic disc hemorrhage in Korea: The Korea National Health and Nutrition Examination Survey. Investig. Ophthalmol. Vis. Sci..

[20] Rural Development Administration (2006). Food Composition Table.

[21] Coleman A.L. (1999). Glaucoma. Lancet.

[22] Jung K.I., Park C.K. (2016). Mental health status and quality of life in undiagnosed glaucoma patients: A nationwide population-based study. Medicine (Baltimore).

[23] Yuki K., Murat D., Kimura I., Ohtake Y., Tsubota K. (2010). Reduced-serum vitamin C and increased uric acid levels in normal-tension glaucoma. Graefes Arch. Clin. Exp. Ophthalmol..

[24] Williams P.A., Harder J.M., Foxworth N.E., Cochran K.E., Philip V.M., Porciatti V., Smithies O., John S.W. (2017). Vitamin B3 modulates mitochondrial vulnerability and prevents glaucoma in aged mice. Science.

[25] Cui X., Chopp M., Zacharek A., Roberts C., Buller B., Ion M., Chen J. (2010). Niacin treatment of stroke increases synaptic plasticity and axon growth in rats. Stroke.

[26] Kaplon R.E., Gano L.B., Seals D.R. (2014). Vascular endothelial function and oxidative stress are related to dietary niacin intake among healthy middle-aged and older adults. J. Appl. Physiol..

[27] Flammer J. (1994). The vascular concept of glaucoma. Surv. Ophthalmol..

[28] Drance S., Anderson D.R., Schulzer M. (2001). Risk factors for progression of visual field abnormalities in normal-tension glaucoma. Am. J. Ophthalmol..

[29] Henry E., Newby D.E., Webb D.J., O’Brien C. (1999). Peripheral endothelial dysfunction in normal pressure glaucoma. Invest. Ophthalmol. Vis. Sci..

[30] Buckley C., Hadoke P.W., Henry E., O’Brien C. (2002). Systemic vascular endothelial cell dysfunction in normal pressure glaucoma. Br. J. Ophthalmol..

[31] Kim E.S., Ko Y.S., Kim J., Matsuda-Inoguchi N., Nakatsuka H., Watanabe T., Shimbo S., Ikeda M. (2003). Food composition table-based estimation of energy and major nutrient intake in comparison with chemical analysis: A validation study in Korea. Tohoku J. Exp. Med..

